# Anti-Bacterial Properties of Cannabigerol Toward *Streptococcus mutans*

**DOI:** 10.3389/fmicb.2021.656471

**Published:** 2021-04-22

**Authors:** Muna Aqawi, Ronit Vogt Sionov, Ruth Gallily, Michael Friedman, Doron Steinberg

**Affiliations:** ^1^Biofilm Research Laboratory, Faculty of Dental Medicine, Institute of Dental Sciences, The Hebrew University of Jerusalem, Jerusalem, Israel; ^2^School of Pharmacy, Institute for Drug Research, The Hebrew University of Jerusalem, Jerusalem, Israel; ^3^The Lautenberg Center for General and Tumor Immunology, Hadassah Medical School, The Hebrew University of Jerusalem, Jerusalem, Israel

**Keywords:** *Streptococcus mutans*, phytocannabinoids, Cannabigerol, dental caries, bacteriostasis

## Abstract

*Streptococcus mutans* (*S. mutans*) is a gram-positive facultatively anaerobic bacterium and the most common pathogen associated with tooth caries. The organism is acid tolerant and can undergo physiological adaptation to function effectively in acid environments such as carious dental plaque. Some cannabinoids have been found to have potent anti-microbial activity against gram-positive bacteria. One of these is the non-psychoactive, minor phytocannabinoid Cannabigerol (CBG). Here we show that CBG exhibits anti-bacterial activities against *S. mutans*. CBG halts the proliferation of planktonic growing *S. mutans*, which is affected by the initial cell density. High-resolution scanning electron microscopy showed that the CBG-treated bacteria become swollen with altered membrane structures. Transmission electron microscopy provided data showing that CBG treatment leads to intracellular accumulation of membrane structures. Nile red, DiOC2(3) and laurdan staining demonstrated that CBG alters the membrane properties, induces membrane hyperpolarization, and decreases the membrane fluidity. CBG-treated bacteria showed increased propidium iodide uptake and reduced calcein AM staining, suggesting that CBG increases the membrane permeability and reduces the metabolic activity. Furthermore, CBG prevented the drop in pH caused by the bacteria. In summary, we present here data showing the mechanisms by which CBG exerts its anti-bacterial effect against *S. mutans*.

## Introduction

Dental caries, also known as tooth decay, is the most common disease of the oral cavity (Krzyściak et al., 2014) and one of the most prevalent chronic human disease worldwide (Robert et al., 2007). Dental caries is a transmissible, complex disease that is caused by prolonged periods of low pH in the mouth, resulting in a net mineral loss from the teeth (Kutsch, 2014). It develops through a complex interaction over time between acid-producing bacteria, fermentable carbohydrates, teeth, and saliva (Krzyściak et al., 2014). More than 700 different bacterial species have been detected in the oral cavity of humans (Larsen and Fiehn, 2017). Among them, is the highly cariogenic bacterium associated with dental caries, *Streptococcus mutans* (*S. mutans*).

*Streptococcus mutans* is a gram-positive, facultatively anaerobic bacterium with acidogenic and aciduric properties (De Sousa et al., 2015; Chu et al., 2016), whose presence within the dental biofilm is an important feature for the establishment and the development of cariogenic dental plaques (Silva et al., 2008). *S. mutans* resides in supragingival plaque, a biofilm formed above the gumline in the oral cavity (Fozo and Quivey, 2004). A major core feature contributing to the organism’s pathogenicity is its ability to transport and metabolize a wide range of nutrients in the hosts’ diet and saliva into organic acids (Lemos et al., 2019). The production of organic acids lowers consequently the pH of the surrounding environment, and over time the drop in pH leads to tooth demineralization and the development of caries. *S. mutans* is capable of surviving in low pH environments (Matsui and Cvitkovitch, 2010) and can metabolize various sugars including glucose, fructose, and sucrose, and acidify the environment to a pH as low as 3.5 (Belli and Marquis, 1991).

The potential use of Cannabis in anti-bacterial therapies has recently emerged. *In vitro* studies have shown that cannabinoids inhibit the growth of Gram-positive bacteria, mostly *Staphylococcus aureus*, with no detectable activity against Gram-negative organisms (Turner and Elsohly, 1981; Appendino et al., 2008). Recently, Blaskovich et al. (2021) observed similar anti-bacterial effect of Cannabidiol (CBD) toward antibiotic-sensitive and antibiotic-resistant *Staphylococcus aureus* with a MIC around 1–5 μg/ml. CBD also had anti-bacterial effect on other gram-positive bacteria (e.g., *Streptococcus pneumoniae* and *Clostridioides difficile)*, as well as a subset of Gram-negative bacteria that includes the “urgent threat” pathogen *Neisseria gonorrhoeae*. Of note, the bacteria didn’t develop resistance to its anti-bacterial activity (Blaskovich et al., 2021).

Cannabigerol (CBG) is a non-psychotropic Cannabis-derived cannabinoid (CB) (Gaoni and Mechoulam, 1964). Several studies support analgesic, anti-depressant, anti-cancer, anti-inflammatory, and anti-hypertensive actions for CBG in mammals (Borrelli et al., 2014; Nachnani et al., 2020). Farha et al. (2020) demonstrated an anti-bacterial activity of CBG against methicillin-resistant *S. aureus* (MRSA). We have previously shown that CBG did not affect the growth of the gram-negative *Vibrio harveyi*, but rather interfered with the quorum sensing system (Aqawi et al., 2020). Here we have studied the anti-bacterial activity of CBG against planktonic growing *S. mutans*.

## Materials and Methods

### Materials

Cannabigerol (CBG) (hemp isolate, 95% purity) was purchased from NC Labs (Czechia) and dissolved in ethanol at a concentration of 10 mg/ml. Respective dilutions of ethanol were used as control.

### Bacterial Growth and Kinetics Studies

Planktonic *S. mutans* UA159 ATCC 700610, *Streptococcus sanguis* 10556, *Streptococcus sobrinus ATCC 27351*, and *Streptococcus salivarius ATCC 25975* were grown overnight at 37°C in 95% air/5% CO_2_ in brain heart infusion broth (BHI, Acumedia, MI, United States) until an OD_600__*nm*_ = 1.2–1.3 was reached (Steinberg et al., 2008). The bacterial cultures were treated with various concentrations of CBG in BHI and respective dilutions of ethanol. Untreated bacteria served as control. For kinetic studies, *S. mutans* with different starting OD_600__*nm*_ (0.1, 0.2, or 0.4) were treated with increasing concentrations of CBG (0, 1.25, 2.5, 5, and 10 μg/ml) and the OD_595__*nm*_ was measured every 30 min for a period of 20 h in a Tecan M200 microplate reader (Tecan Trading AG, Switzerland) at 37°C.

### Colony Forming Units (CFU)

Colony forming units assay was performed after different incubation time (0, 2, 4, 6, 8, and 24 h) with CBG (0, 2.5, 5, and 10 μg/ml). 10-fold serial dilutions of the untreated and treated samples were prepared by repeatedly transferring 100 μl from one sample to another tube containing 900 μl PBS. After vigorous vortex, 100 μl of the bacterial suspensions were spread on BHI agar plates and incubated overnight at 37°C in the presence of 5% CO_2_. After incubation, the number of colonies was counted using the ImageJ software (Breed and Dotterrer, 1916). The following equation was used to calculate the CFU per ml in the original sample:

CFU per ml = Number of colonies × dilution factor × 1/volume seeded on the plates.

### High Resolution Scanning Electron Microscopy (HR-SEM)

Planktonic growing *S. mutans* of OD_600__*nm*_ = 0.1 was treated with different concentrations of CBG (0, 2.5, 5, and 10 μg/ml) for 4 h. At the end of incubation, the bacteria were washed twice with PBS, fixed in 4% glutaraldehyde for 40 min and washed in double distilled water. The specimens were then mounted on glass pieces, sputter coated with iridium and visualized using a Magellan^TM^ 400L High-Resolution Scanning Electron Microscope (FEI Company, Holland) (Brandwein et al., 2016). Images were captured randomly from 4 to 5 different areas. The lengths and width of the bacteria were measured using the ImageJ software. 200 bacteria were measured for each treatment group from 8 to 9 independent high magnification images.

### Transmission Electron Microscopy (TEM)

Untreated and CBG (10 μg/ml)-treated *S. mutans* at an OD_600__*nm*_ = 0.1 were incubated for 4 h. At the end of incubation, the bacteria were washed twice with PBS, followed by an overnight fixation in 2% formaldehyde and 2.5% glutaraldehyde in 0.1 M sodium cacodylate buffer, pH 7.4. The fixed bacteria were rinsed four times 10 min in 0.1 M cacodylate buffer and stained with 1% osmium tetroxide and 1.5% potassium ferricyanide in 0.1 M cacodylate buffer for 1 h. The bacteria were then washed four times in cacodylate buffer followed by dehydration in increasing concentrations of ethanol consisting of 30%, 50%, 70%, 80%, 90%, 95% for 10 min each step, followed by three times 20 min in 100% anhydrous ethanol, and two times 10 min in propylene oxide. Each step was followed by centrifugation at 15,000 *g* for 8 s. Following dehydration, the cells were infiltrated with increasing concentrations of Agar 100 resin in propylene oxide, consisting of 25, 50, 75, and 100% resin for 16 h each step. The bacteria were then embedded in fresh resin and let polymerize in an oven at 60°C for 48 h. Embedded bacteria in blocks were sectioned with a diamond knife on a Leica Reichert Ultracut S microtome and ultrathin sections (80 nm) were collected onto 200 mesh, thin bar copper grids. The sections on grids were sequentially stained with uranyl acetate and lead citrate for 10 min each and viewed with Tecnai 12 TEM 100 kV (Phillips, Eindhoven, Netherlands) equipped with MegaView II CCD camera and Analysis^®^ version 3.0 software (Soft Imaging System GmbH, Münster, Germany).

### Microbial Cell Viability Assay

The luminescent BacTiter-Glo^TM^ kit (Promega) was used to quantify the ATP levels in untreated and CBG-treated samples. Briefly, 100 μl of each sample (CBG = 0, 2.5, 5, 10, and 20 μg/ml for 2 h) was mixed with 100 μl of the reagent in 96-flat bottom plates (Greiner Bio-One, μClear white clear bottom plates), and after mixing for 5 min on an orbital shaker, the luminescence was recorded using the M200 Tecan plate reader. ATP level was calculated in comparison to the control using the following equation: (sample luminescence/control luminescence) × 100.

### Membrane Permeability Assay

Changes in the bacterial cell membrane permeability was assessed by propidium iodide (PI) (Sigma) uptake and the metabolic activity by calcein AM (BioLegend) staining essentially as described (Cho and Lee, 2011; Ohsumi et al., 2015). PI enters only membrane-compromised cells and fluoresces in the red spectrum when binding to nucleic acids within the cells (Veerman et al., 2004). On the other hand, calcein AM diffuses passively into the cytoplasm, where it is converted into green fluorescent calcein via native esterases. Calcein fluorescence is retained in live cells but leaks out when the plasma membrane is compromised. An overnight culture of *S. mutans* was resuspended in BHI to an OD_600__*nm*_ = 0.3. The bacteria were then treated with different concentrations of CBG (0, 1.25, 2.5, 5, 8, and 10 μg/ml) for 2 h at 37°C. At the end of incubation, the bacteria were stained with 10 μg/ml PI and 10 μg/ml calcein AM for 20 min at 37°C, followed by flow cytometry (BD LSR-Fortessa flow cytometer, BD Biosciences) using the 488 nm excitation laser and collecting the data using the red and green filters, respectively.

### Laurdan Membrane Fluidity Assay

The membrane fluidity of *S. mutans* was measured using laurdan (AnaSpec, Fremont, CA, United States) essentially as described (Bessa et al., 2019). Laurdan is a fluorescence probe that intercalates into the membrane bilayer and displays an emission wavelength shift depending on the amount of water molecules in the membrane (Wenzel et al., 2018). *S. mutans* (OD_600_ = 0.3 nm) was treated with different concentrations of CBG (0, 4, 6, 8, 10, and 20 μg/ml) at 37°C for 2 h and then incubated with 10 μM laurdan for 10 min at room temperature in the dark. An unstained sample served as control. Thereafter, the samples were washed four times in PBS containing 1% glucose and 1% DMSO (PBSGD) and resuspended in 1 ml of PBSGD. 200 μl of each sample were added to each well of a μClear black 96-well plate (Greiner Bio-One, Frickenhausen, Germany) and the fluorescence analyzed at 30°C in the M200 Tecan plate reader with an excitation at 350 nm and an emission scan from 400 to 600 nm. The laurdan Generalized Polarization (GP) was calculated using the following equation: GP = (I_440_−I_490_)/(I_440_ + I_490_) where I_440_ and I_490_ are fluorescence intensities at 440 and 490 nm, respectively.

### Nile Red Membrane Staining

Control bacteria (OD_600__*nm*_ = 0.3) or bacteria that have been exposed to CBG (0, 1.25, 2.5, 5, and 10 μg/ml) for 2 h, were stained with 10 μg/ml Nile red (APExBIO, Boston, MA, United States) and 4′,6-Diamidine-2′-phenylindole dihydrochloride (DAPI) (Sigma) for 30 min at 37°C (Sugimoto et al., 2017). After washing the cells in PBS, the bacteria were analyzed on flow cytometry (LSR-Fortessa flow cytometer, BD Biosciences) using the 561 nm yellow-green laser excitation and collecting the data using the 635 nm filter.

### Membrane Potential (MP)

The membrane potential of *S. mutans* was measured using cationic dye 3,3′-diethyloxacarbocyanine iodide (DiOC_2__(__3__)_; Molecular Probes, Eugene, OR, United States) by flow cytometry according to the manufacturer’s instructions. DiOC_2__(__3__)_ exhibits green fluorescence in all bacterial cells, but the fluorescence shifts toward red emission at larger membrane potential. An overnight culture of *S. mutans* was resuspended in PBS to an OD_600__*nm*_ = 0.3 and exposed to different concentrations of CBG (0, 2.5, 5, and 10 μg/ml) and 30 μM DiOC_2__(__3__)_ for 30 min at room temperature. The samples were analyzed by flow cytometry (LSR-Fortessa flow cytometer) using the 488 nm excitation laser and collecting the data using the green and red filters.

### pH Measurements

*Streptococcus mutans* of OD_600__*nm*_ = 0.1 was treated with different concentrations of CBG (0, 2.5, 5, and 10 μg/ml) and incubated at 37°C for 24 h. At various time points, the pH of the samples was measured using pH-indicator strips (MColorpHast, Merck KGaA, Darmstadt, Germany).

### Drop Plate Method

Drop plate method was performed after different incubation time (0, 2, 4, 6, 8, and 24 h). Here, an agar plate was divided into sectors. In each sector, one drop of 10 μl from untreated and CBG- treated bacteria (0, 2.5, 5, and 10 μg/ml) was inoculated on the surface of the agar, and thereafter the plates were incubated upside down at 37°C overnight.

### Statistical Analysis

Experiments were performed independently three times in triplicates and the data were analyzed statistically using Student’s *t* test in Microsoft Excel, with a *p* value of less than 0.05 considered significant.

## Results

### CBG Halts the Proliferation of Planktonic *S. mutans*

**FIGURE 1:**
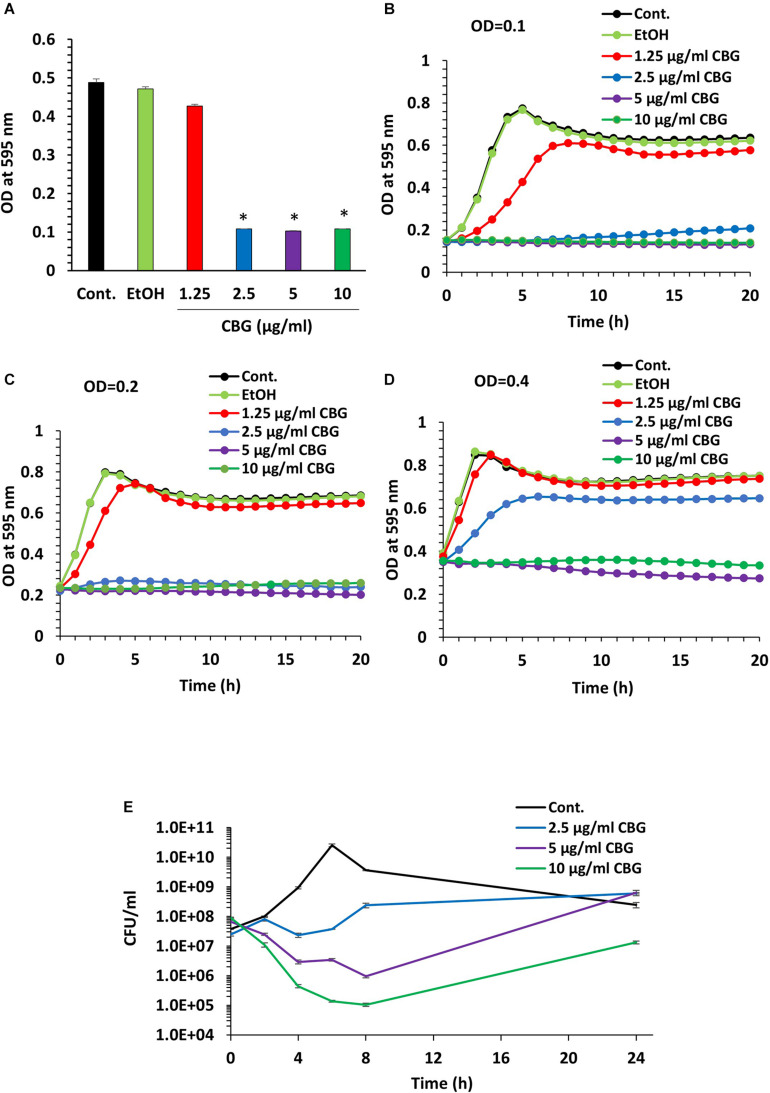
CBG halts the proliferation of planktonic growing *S. mutans*. **(A)** The viability of *S. mutans* after a 24 h incubation with increasing doses of CBG (0–10 μg/ml) as measured by OD_595__*nm*_. *n* = 3; *p < 0.05. **(B–D)** A kinetic study of the planktonic growth of *S. mutans* in the presence of increasing concentrations of CBG (0–10 μg/ml) at starting OD_600__*nm*_ = 0.1 **(B)**, 0.2 **(C)**, and 0.4 **(D)**. **(E)** The colony forming units of untreated and CBG (0–10 μg/ml)-treated bacteria at various time points. *n* = 3.

We initially analyzed the effect of CBG on *S. mutans* viability. For this purpose, *S. mutans* was exposed to increasing concentrations of CBG, and the OD_595__*nm*_ monitored after 24 h incubation (Figure 1A). We found that CBG inhibited in a dose-dependent manner the planktonic growth of *S. mutans* with a MIC of 2.5 μg/ml (Figure 1A). CBG had also an anti-bacterial effect toward *S. sanguis*, *S. sobrinus*, and *S. salivarius* (Table 1). To get a better insight into the CBG effect on the bacterial growth, kinetic growth studies were performed using different initial bacterial densities (OD_600__*nm*_ = 0.1–0.4) in the presence of increasing concentrations of CBG (Figures 1B–D). When starting with an OD_600__*nm*_ = 0.1 or 0.2, CBG treatment arrested the growth of *S. mutans* at a concentration of 2.5, 5, and 10 μg/ml, whereas 1.25 μg/ml only delayed the onset of the bacterial log growth phase (Figures 1B,C). When increasing the initial OD_600__*nm*_ to 0.4, CBG at 1.25 μg/ml had no effect, CBG at 2.5 μg/ml showed a delayed onset of the log-phase, while CBG at 5 and 10 μg/ml still retained their growth inhibition effect without any sign of recovery even after 24 h (Figure 1D). The OD of the bacteria treated with 10 μg/ml CBG was even 25% lower after 24 h than the initial OD (Figure 1D). Altogether, these data suggest that the growth-inhibitory effect of CBG is affected by the initial cell density, and higher CBG concentrations are needed to achieve full growth inhibitory effect at higher densities.

**TABLE 1:** The minimal inhibitory concentration (MIC) of CBG toward four different oral bacteria.

Type of bacteria	MIC (CBG)
*Streptococcus mutans*	2.5 μg/ml
*Streptococcus sanguis*	1 μg/ml
*Streptococcus sobrinus*	5 μg/ml
*Streptococcus salivarius*	5 μg/ml

To examine whether CBG is bacteriostatic or bactericidal, 10 μl from each sample was applied on BHI agar plates at different time points during the 24 h incubation period. Bacterial growth was observed at all tested CBG concentrations (Supplementary Figure 1), suggesting that CBG is bacteriostatic, and the bacterial growth can be recovered when CBG is removed. To quantify the growth inhibitory effect of CBG, we counted the colony-forming units (CFUs) at different time points during the 24 h incubation (Figure 1E). Again, the growth arrest phenomenon was observed with the CBG-treated cells exhibiting a multitude lower cell counts/ml than their control counterparts (Figure 1E). As expected, the control bacteria continued to grow, reaching a maximum number of live bacteria after 6 h followed by a decline during the next 18 h (Figure 1E). Bacteria exposed to CBG at 2.5 μg/ml showed an initial growth arrest, that was followed by a slow growth rate after 6 h (Figure 1E). At 5 and 10 μg/ml, CBG resulted in a gradual drop in the number of viable bacteria during the first 8 h, followed by a recovery of surviving bacteria (Figure 1E). 5 and 10 μg/ml CBG led to a respective 98.5% and 99.9% reduction of viable bacteria at 8 h (Figure 1E). This finding indicates that CBG at the higher concentrations have a bactericidal effect, in addition to having a bacteriostatic effect.

### HR-SEM Images Show Altered Membrane Structures of CBG-Treated Bacteria

**FIGURE 2:**
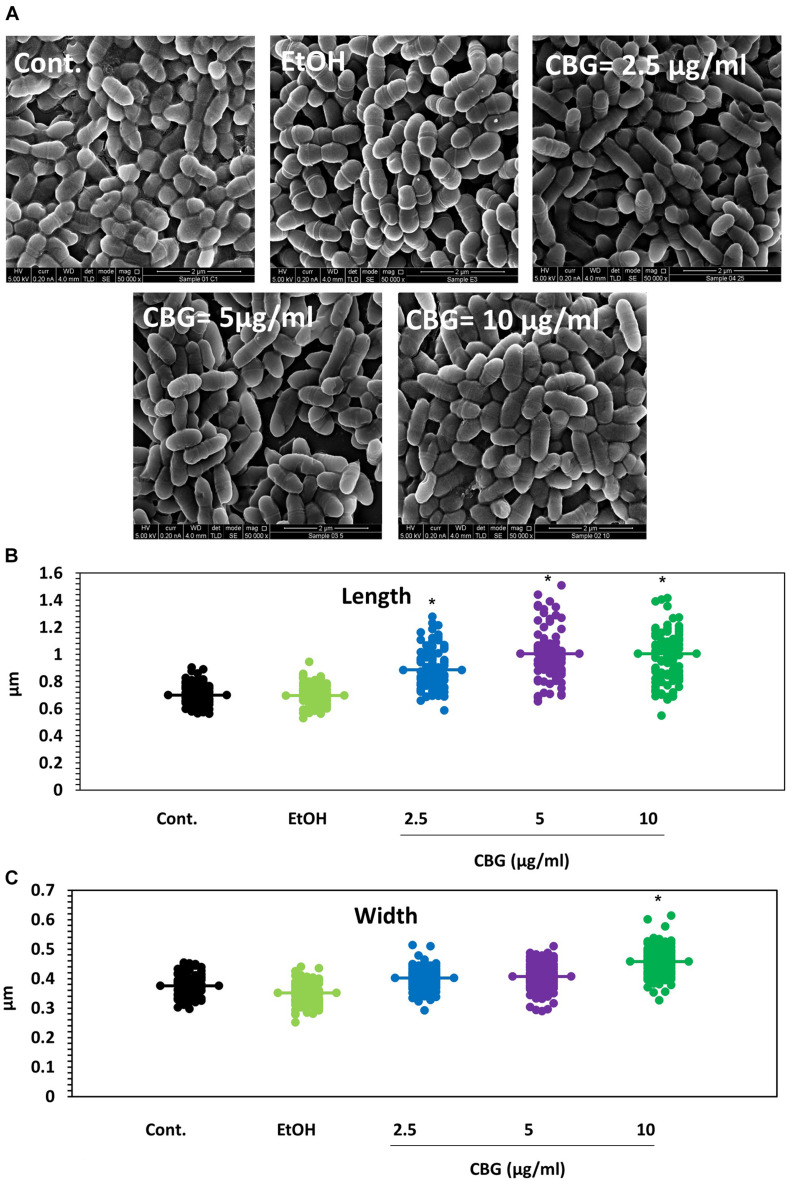
CBG alters the membrane structure and the size of *S. mutans*. **(A)** HR-SEM images (x50000) of control bacteria or bacteria treated with different concentrations of CBG (0–10 μg/ml) for 4 h. **(B)** The average length of untreated and CBG (0–10 μg/ml)-treated bacteria. *n* = 200; *p < 0.05. **(C)** The average width of untreated and CBG (0–10 μg/ml)-treated bacteria. *n* = 200; *p < 0.05.

To understand in more depth the effects of CBG, control and CBG-treated bacteria (2.5, 5, and 10 μg/ml for 4 h) were visualized under a high-resolution scanning electron microscope (HR-SEM) (Figure 2A). The CBG-treated bacteria appear longer at the average in comparison to control bacteria and folded membrane structures could often be seen (Figure 2A). At 10 μg/ml CBG, many of the bacteria seem to be swollen (Figure 2A). When the length (Figure 2B) and the width (Figure 2C) were measured using the ImageJ software, the control sample showed an average length of 0.7 μm and an average width of 0.37 μm, while bacteria treated with CBG appear larger reaching an average length of 1 μm and an average width of 0.46 μm at 10 μg/ml.

### CBG Leads to Intracellular Accumulation of Membrane Structures

**FIGURE 3:**
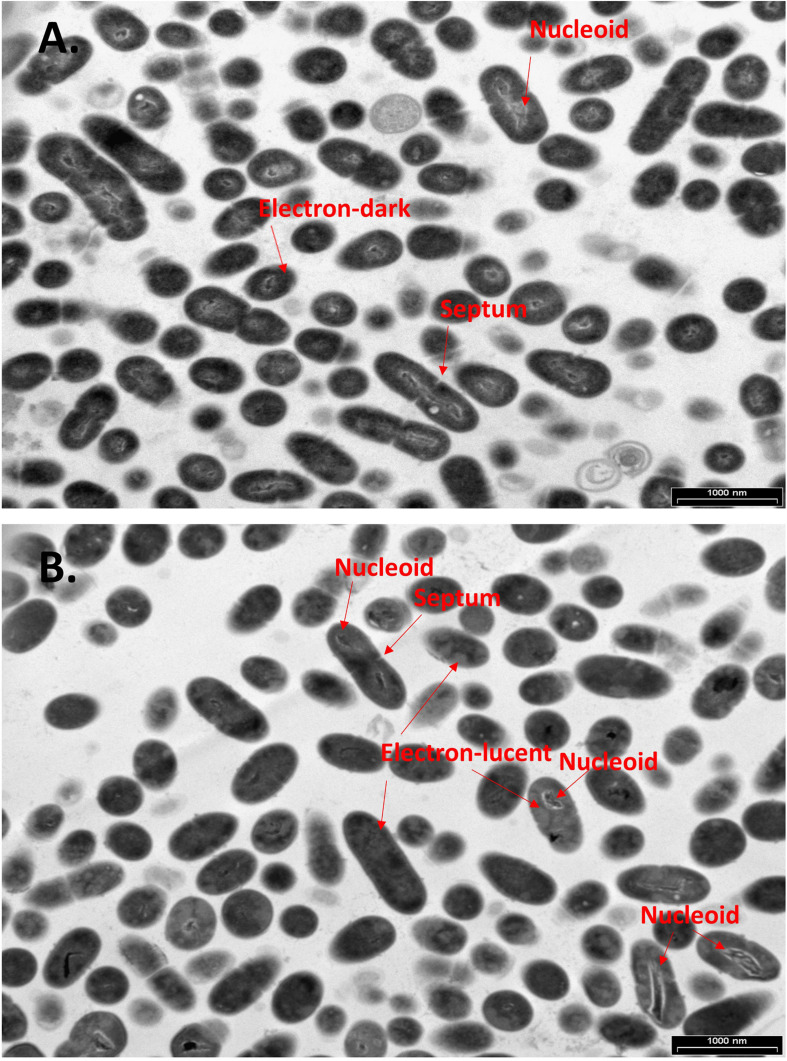
CBG alters the morphology of *S. mutans.* **(A,B)** Panoramic TEM images (x9700) of control **(A)** and 4 h CBG (10 μg/ml)-treated **(B)** bacteria. The arrows point to the nucleoids, the electron-dense and electron-lucent areas, and the invagination septum in control and CBG-treated *S. mutans*.

**FIGURE 4:**
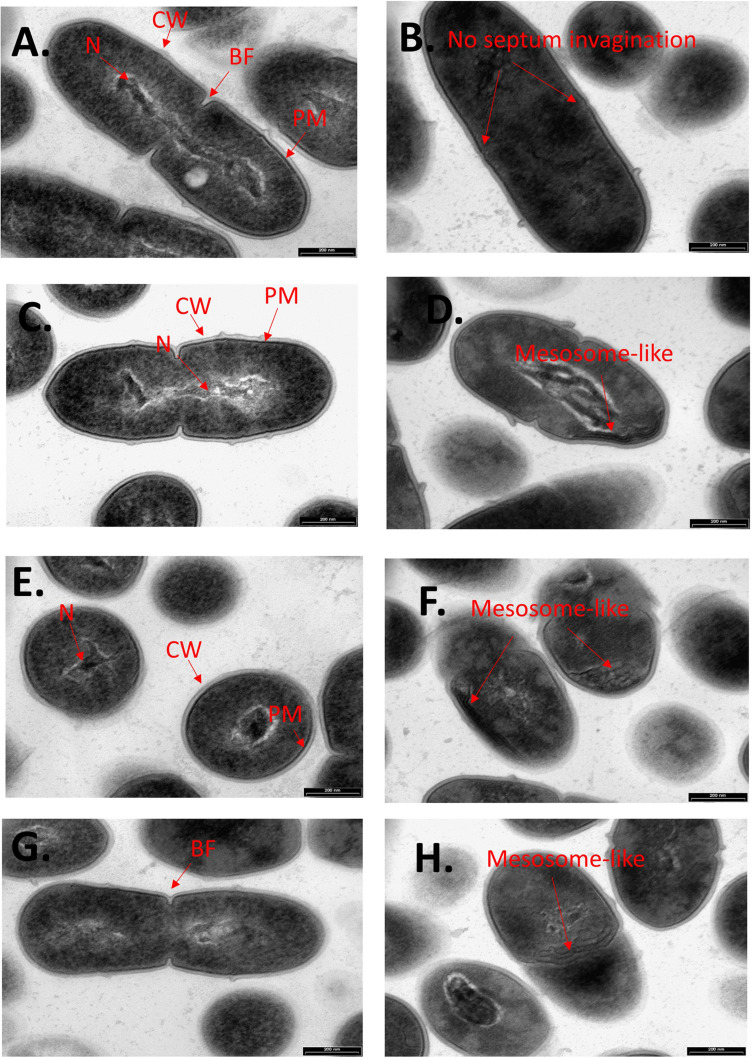
CBG alters the morphology of *S. mutans.* Higher magnification TEM images (x59000) of control **(A,C,E,G)** and 4 h CBG (10 μg/ml)-treated **(B,D,F,H)** *S. mutans*. The arrows show the cell wall (CW), cell membrane (PM), nucleus (N), the invagination septum (BF-Binary fission), and the mesosome-like structures.

To further examine the effect of CBG on *S. mutans*, the morphology of untreated and CBG (10 μg/ml for 4 h)-treated bacteria was studied by transmission electron microscopy (TEM) (Figures 3, 4). In the panoramic low magnification images (Figure 3A), we can clearly see that most of the control bacteria show structured nucleoids symmetrically distributed eccentrically in the dividing bacteria. The cytoplasm of the control bacteria appears homogenously with electron-dense material and there are several dividing bacteria with initial membrane invagination for septum formation (Figure 3A). The nucleoids of CBG-treated bacteria showed either a distorted structure or could not been observed (Figure 3B). The CBG-treated bacteria barely showed any sign of septum invagination (Figure 3B). Strikingly, the cytoplasm of the CBG-treated bacteria showed accumulation of electron-lucent, bright material (Figure 3B). At higher magnification of control bacteria, we could clearly distinguish between the intact and well-defined cell wall (CW), plasma membrane (PM), and cytoplasmic space with a central nucleoid (N) containing the circular DNA (Figures 4A,C,E,G). In Figures 4A,C, control bacteria with initial invagination of the plasma membrane can be observed. When treated with CBG, there is a disturbance of the bacterial plasma membrane and mesosome-like structures of bacterial membranes are observed within the bacteria (Figures 4B,D,F,H). In Figure 4B, the CBG-treated bacteria appeared longer than usual with no sign of septum invagination.

### The Effect of CBG on ATP Levels in *S. mutans*

**FIGURE 5:**
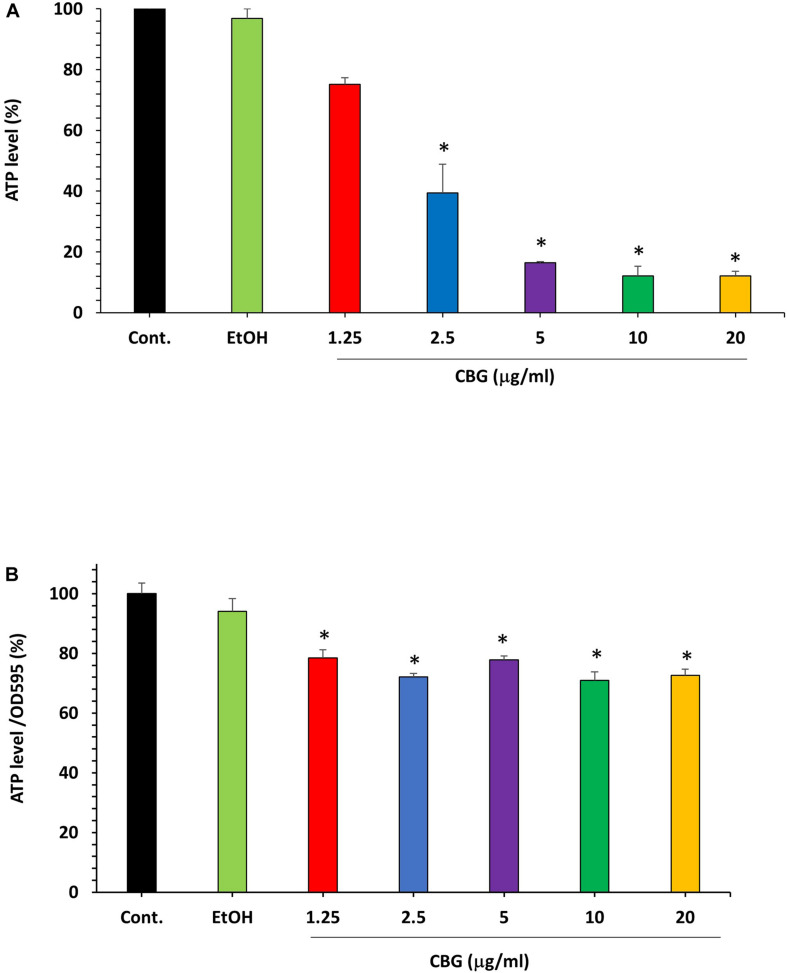
The effect of CBG on the ATP levels in *S. mutans*. **(A)** The percentage ATP levels in *S. mutans* treated with various concentrations of CBG (0–20 μg/ml) for 2 h in comparison to control. *n* = 3; *P < 0.05. **(B)** The percentage of the ATP/OD_595__*nm*_ normalized levels in *S. mutans* treated with various concentrations of CBG (0–20 μg/ml) for 2 h in comparison to control. *n* = 3; *p < 0.05.

The microbial cell viability assay was performed to analyze the effect of CBG on the ATP levels in *S. mutans*. For that purpose, *S. mutans* was exposed to different concentrations of CBG and the amount of ATP was measured after 2 h. The percentage reduction in ATP level was calculated in comparison to untreated and ethanol-treated samples. According to these data, CBG at 1.25 μg/ml reduced the ATP level to 75.0 ± 2.2% (Figure 5A; *p* < 0.05). When increasing the CBG concentrations to 5, 10, and 20 μg/ml, the ATP levels were reduced to 16.4 ± 0.3%, 12.1 ± 3.1%, and 12.0 ± 1.5%, respectively. Ethanol barely had any effect. When the ATP level of each sample was divided by its own OD_595__*nm*_ to normalize the ATP level to cell density (Figure 5B), there was only a slight reduction in the relative ATP level of around 20–30%. Thus, the major reduction in the ATP levels in Figure 5A was due to the reduced number of bacteria and the net ATP level per cell is only slightly lower following CBG treatment.

### CBG Alters the Membrane Properties of *S. mutans*

**FIGURE 6:**
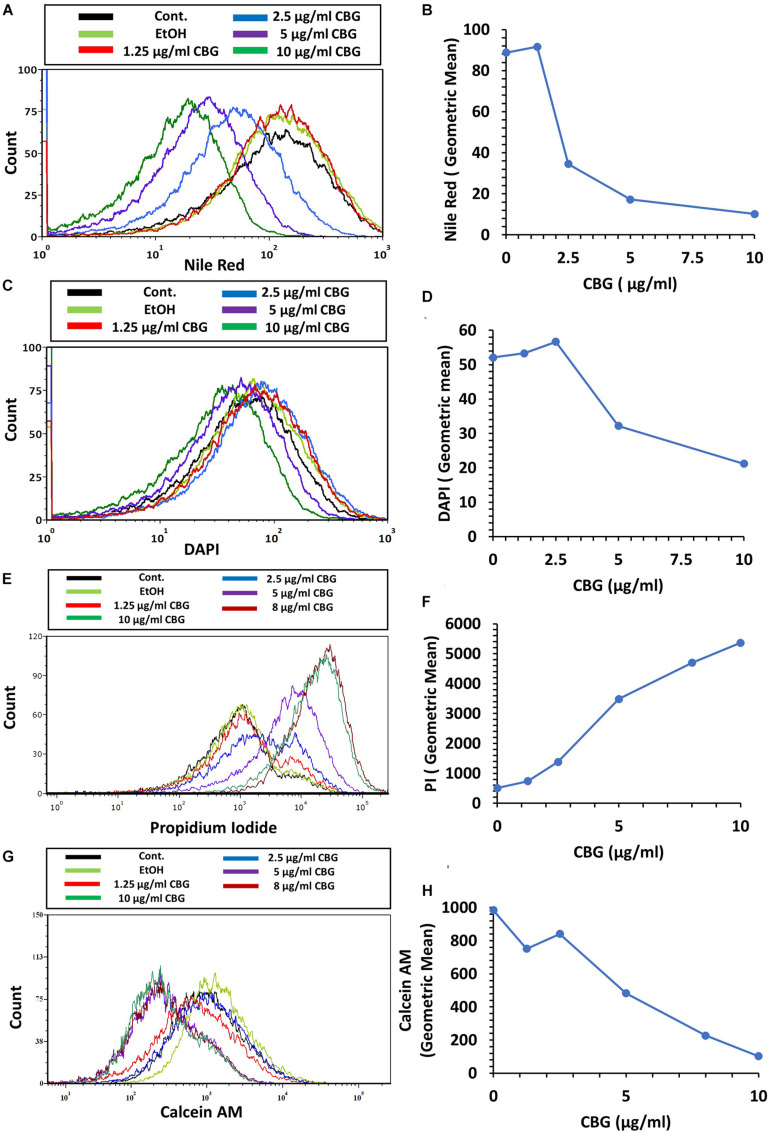
CBG alters the membrane properties of *S. mutans.* **(A,B)** The fluorescence intensity of Nile Red membrane staining of control and 2 h CBG (0–10 μg/ml)-treated bacteria as determined by flow cytometry. **(C,D)** The fluorescence intensity of DAPI staining of control and 2 h CBG (0–10 μg/ml)-treated bacteria as determined by flow cytometry. **(E,F)** Flow cytometry of PI-stained *S. mutans* that have been treated with different CBG concentrations (0–10 μg/ml) for 2 h. **(G,H)** Flow cytometry of Calcein AM-stained *S. mutans* that have been treated with different CBG concentrations (0–10 μg/ml) for 2 h. **(A,C,E,G)** are the histograms of flow cytometry. **(B,D,F,H)** present the geometric mean of the flow cytometry data as a function of CBG concentration.

We used the hydrophobic fluorescent probe Nile Red to stain the bacterial membrane of control and bacteria treated with various concentrations of CBG for 2 h. Despite more membrane-like structures within the CBG-treated bacteria observed with TEM (Figures 4D,F,H), there was a dose-dependent decrease in Nile Red staining (Figures 6A,B). These data suggest that CBG leads to a reduction in the membrane mass or it causes a less hydrophobic membrane. Simultaneous staining of the DNA of the bacteria by DAPI shows only a slight reduction in DNA content with increasing concentrations of CBG (Figures 6C,D).

### CBG Increases the Membrane Permeability of *S. mutans*

To study the effect of CBG on membrane permeability, untreated and bacteria treated with various concentrations of CBG for 2 h were incubated with propidium iodide (PI) and calcein AM for 20 min before analyzing the cells by flow cytometry. CBG treatment led to a dose-dependent increase in PI uptake (Figures 6E,F), while calcein AM staining was concomitantly reduced (Figures 6G,H). The increase in PI uptake after CBG treatment indicates an increase in membrane permeability, while the reduced calcein AM staining is a combined effect of reduced metabolic activity and leakage of calcein out of the cells.

### CBG Causes Immediate Membrane Hyperpolarization in *S. mutans*

**FIGURE 7:**
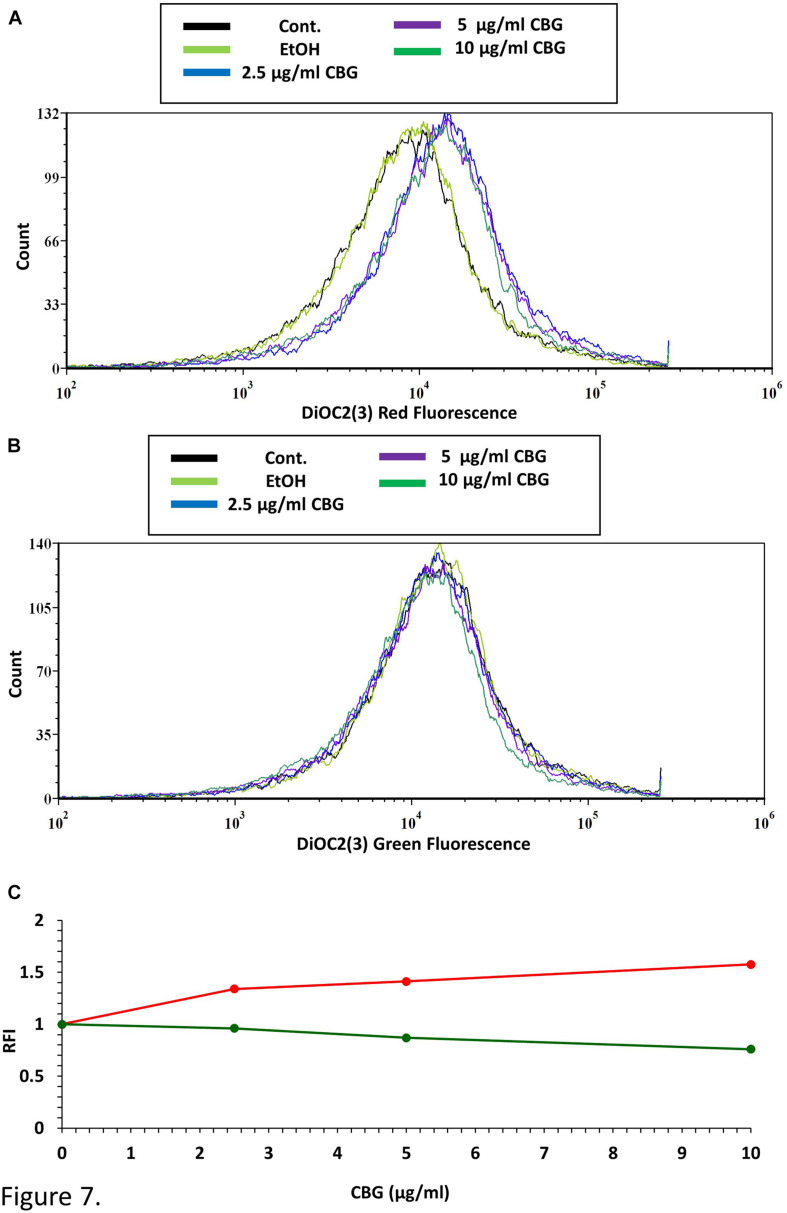
CBG causes membrane hyperpolarization in *S. mutans*. **(A)** The red fluorescence of DiOC2(3)-stained *S. mutans* that have been exposed to different CBG concentrations (0–10 μl/ml) for 30 min. **(B)** The green fluorescence of DiOC2(3)-stained *S. mutans* that have been exposed to different CBG concentrations (0–10 μl/ml) for 30 min. **(C)** The relative fluorescence intensity (RFI) of the red (red lines) and green (green lines) fluorescence of the samples presented in **(A,B)**.

To test the direct effect of CBG on the membrane potential of *S. mutans*, bacteria were loaded with the membrane-potential-sensitive cyanine dye DiOC_2__(__3__)_, and then treated with increasing concentrations of CBG and the green/red fluorescence intensity immediately monitored by flow cytometry. As the concentration of the CBG increases, the red fluorescence intensities were increased by 150% (Figures 7A,C and Supplementary Figure 2), while the green fluorescence intensities were decreased by 25% (Figures 7B,C and Supplementary Figure 2), indicating that CBG causes hyperpolarization of the membranes. It is noteworthy to mention that the changes in membrane potential is an immediate effect of CBG, suggesting that CBG acts on the plasma membrane.

### CBG Reduces the Membrane Fluidity of *S. mutans*

**FIGURE 8:**
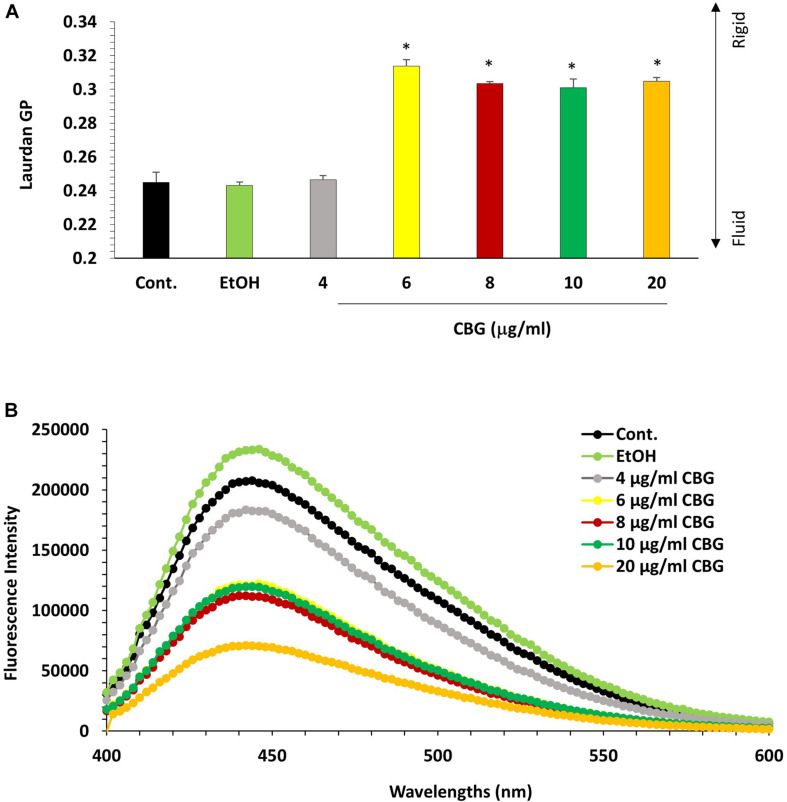
CBG reduces *S. mutans* membrane fluidity. **(A)** Laurdan generalized polarization (GP) values in *S. mutans* treated with various concentrations of CBG (0–20 μg/ml) for 2 h. *n* = 3; *p < 0.05. **(B)** Fluorescence intensity scan of laurdan stained *S. mutans* that have been treated with different CBG concentrations (0–20 μg/ml) or EtOH for 2 h.

The effect of CBG on the bacterial membrane fluidity was studied by staining the untreated and CBG-treated bacteria with laurdan, which is a fluorescent probe that intercalates into the membrane bilayer and displays an emission wavelength shift from 440 to 490 nm depending on the amount of water molecules in the membrane. An inverse relationship exists between the laurdan generalized polarization (GP) values and the degree of membrane fluidity (Colalto, 2018). Also, the higher the laurdan staining, the higher is the fluidity. CBG treatment caused an increase in laurdan GP values (Figure 8A), suggesting a more rigid membrane. The reduced membrane fluidity is further supported by the observation that CBG caused a dose-dependent reduction in laurdan incorporation (Figure 8B).

### CBG Treatment Prevents the Drop in pH Caused by *S. mutans*

**FIGURE 9:**
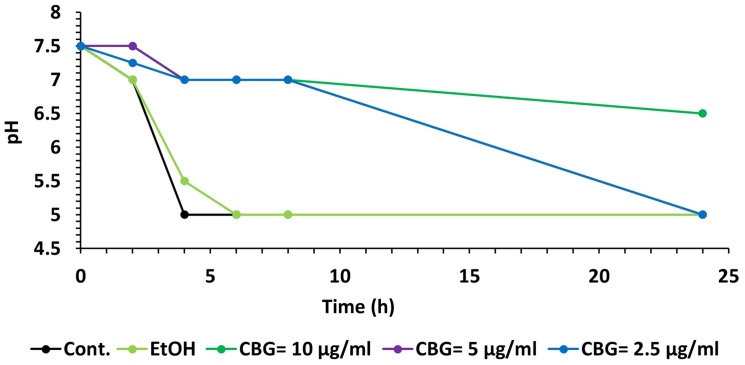
CBG prevents the decrease in pH caused by *S. mutans*. A kinetic change in the pH values of the medium of untreated and CBG (0–10 μg/ml)-treated *S. mutans*.

A kinetic study of the pH level in the *S. mutans* culture media showed that CBG was able to maintain the pH at 7 for at least 8 h at all tested concentrations (Figure 9). After 24 h, the pH in the 2.5 and 5 μg/ml CBG samples had reached five similarly to the control samples, while 10 μg/ml CBG still managed to prevent the acidification (pH = 6.5) (Figure 9). The latter correlates with the prevention of bacterial growth with this concentration.

## Discussion

Recently, the anti-bacterial properties of several medicinal plants have been explored (Colalto, 2018). *Cannabis sativa* is an herbaceous plant that has been used for millennia for medicinal and recreational purposes. Cannabis is undoubtedly one of the most widely used illicit drugs (Bewley-Taylor, 2002). Natural products derived from Cannabis and their analogs have been screened for anti-microbial properties, in the quest to discover new anti-infective agents. Several cannabinoids have been found to have potent anti-microbial activity against Gram-positive pathogens such as MRSA isolates (Karas et al., 2020). Cannabigerol (CBG) is one of the phytocannabinoids present in *Cannabis sativa* L. that has attracted pharmacological interest because it is non-psychotropic and is abundant in some industrial hemp varieties (Navarro et al., 2018).

Bacteria belonging to the genus *Streptococcus* are the first inhabitants of the oral cavity, which can be acquired right after birth and thus play an important role in the formation of the oral microbiota (Abranches et al., 2019). In the oral cavity, many microorganisms have been found to be associated with dental caries, among them *S. mutans* is considered the most cariogenic bacteria (Loesche, 1986). Therefore, the inhibition of *S. mutans* is a key objective in the prevention of dental caries.

In the present study, we aimed to investigate the anti-bacterial properties of CBG against planktonic growing *S. mutans*. We demonstrated that CBG exerts a bacteriostatic effect at a concentration of 2.5 μg/ml and the growth-inhibitory effect of CBG is affected by the initial cell density. At the higher concentrations of 5–10 μg/ml, CBG had a bactericidal effect as shown by 98.5–99.9% reduction of viable bacteria after 8 h. This is further manifested by the increased uptake of PI, that only penetrates bacteria with perforated membrane. The latter observation suggests that CBG increases membrane permeability. The dose-dependent reduction in Calcein AM staining with increasing concentrations of CBG points to enhanced membrane leakage. These findings go along with the observation by Blaskovich et al. (2021) that the primary anti-bacterial action of CBD is destruction of the membrane. Also, Farha et al. (2020) provided evidence that CBG acts by perturbing the plasma membrane and gram-negative bacteria can be sensitized to CBG after permeabilization of the outer membrane by polymyxin B. The reduced Calcein AM staining might also indicate reduced metabolic activity, but the ATP levels per bacteria were only slightly reduced by CBG after a 2 h incubation.

Kinetic growth studies showed that CBG at 1.25 μg/ml delayed the initiation of the bacterial log growth phase, while CBG at higher concentrations retained their growth inhibition effect with a sign of recovery after 24 h. This observation indicates that CBG impedes cell division. This is further confirmed by TEM, where the number of septal invaginations is strongly reduced in CBG-treated bacteria in comparison to control bacteria. The TEM images also showed that CBG leads to a disturbance of the plasma membrane with an accumulation of mesosome-like membrane structures within the bacteria. A similar appearance of mesosome-like structures was observed by Xiao et al. (2020), when MRSA was treated with an α/β chimeric polypeptide molecular brush. HR-SEM images confirmed altered bacteria membrane structures following CBG treatment. The bacteria appeared swollen, became longer and wider at the average after a short period of 4 h incubation with CBG. Moreover, the CBG-treated bacteria showed irregular folded membrane structures.

Since the TEM images showed prominent changes in the membrane structures of *S. mutans* following CBG treatment, we performed a series of experiments to test the effect of CBG on the cell membrane. We initially used Nile Red to stain the membranes. Our data showed a dose-dependent decrease in Nile Red staining, suggesting that CBG reduces the total membrane mass. An alternative explanation for the reduced Nile Red staining could be CBG-induced alterations in membrane polarity which affects the fluorescence intensity of this dye (Sackett and Wolff, 1987). Nile red in polar membranes fluoresces dark red, while the color shifts to yellow-gold emission in neutral lipids. In a parallel assay using the membrane dye laurdan, CBG treatment reduced the incorporation of this compound in a dose-dependent manner indicative for a more rigid membrane. On top of these findings, we observed that CBG caused immediate membrane hyperpolarization in *S. mutans* suggesting an effect on ion channels. Altogether, our data suggest that CBG alters the cell membrane properties of *S. mutans*.

Membrane fluidity is a key parameter of bacterial membranes that undergoes quick adaptation in response to environmental challenges and has recently emerged as an important factor in the anti-bacterial mechanism of membrane-targeting antibiotics. Assessing changes in the overall membrane fluidity and formation of membrane microdomains is therefore pivotal to understand both the functional organization of the bacterial cell membrane as well as the antibiotic mechanisms (Wenzel et al., 2018). To withstand the acidification of its environment, *S. mutans* shifts its lipid profile from saturated fatty acid (rigid) at pH = 7 to a more unsaturated (fluid) fatty acid at pH = 5 (Quivey et al., 2000). Thus, the effect of CBG on *S. mutans* membrane properties might contribute to its anti-bacterial effect by preventing the necessary adaptation to an acid environment. We observed that CBG prevented the drop in pH caused by *S. mutans.* CBG was able to maintain the pH at 7 for at least 8 h at all tested concentrations. The maintenance of pH could be related to the reduced proliferation of the bacteria.

In summary, the present study demonstrates an anti-bacterial effects of the Cannabis component CBG toward the cariogenic bacteria *S. mutans*. CBG acts at several levels: (1) It exerts a bacteriostatic effect that is affected by the initial bacterial cell density. (2) It affects the membrane structure and causes intracellular accumulation of mesosome-like structures. (3) It causes immediate membrane hyperpolarization. (4) It reduces the membrane fluidity. (5) It increases the membrane permeability. (6) It prevents the drop in pH caused by *S. mutans*, thereby preventing its cariogenic property. The interference of CBG with the caries causative *S. mutans* may provide a novel innovative way to combat dental caries.

## Data Availability Statement

The raw data supporting the conclusions of this article will be made available by the authors, without undue reservation.

## Author Contributions

MA, RS, RG, MF, and DS conceived the idea. MA designed and performed the experiments, analyzed the data, and wrote the manuscript with RS and DS. All authors contributed to the article and approved the submitted version.

## Conflict of Interest

The authors declare that the research was conducted in the absence of any commercial or financial relationships that could be construed as a potential conflict of interest.

## Supplementary Material

Supplementary Figure 1 Drop method to detect bacterial growth of *S. mutans* on BHI-agar plates after treatment with different concentrations of CBG (0–10 μg/ml) at various time points. At the end of incubation, 10 μl of each sample was applied in triplicates on BHI agar plates and incubated overnight at 37°C.

Supplementary Figure 2 Flow cytometry dot plots of DiOC2(3)-stained *S. mutans* that have been exposed to different CBG concentrations (0–10 μl/ml) for 30 min. The *Y*-axis shows the red fluorescence, while the *X*-axis shows the green fluorescence.
