# Supplementary figures and images for: Anti-Bacterial Properties of Cannabigerol Toward Streptococcus mutans
Source: Front Microbiol. 2021 Apr 22;12:656471. doi: 10.3389/fmicb.2021.656471 (PMC8100047; doi:10.3389/fmicb.2021.656471)

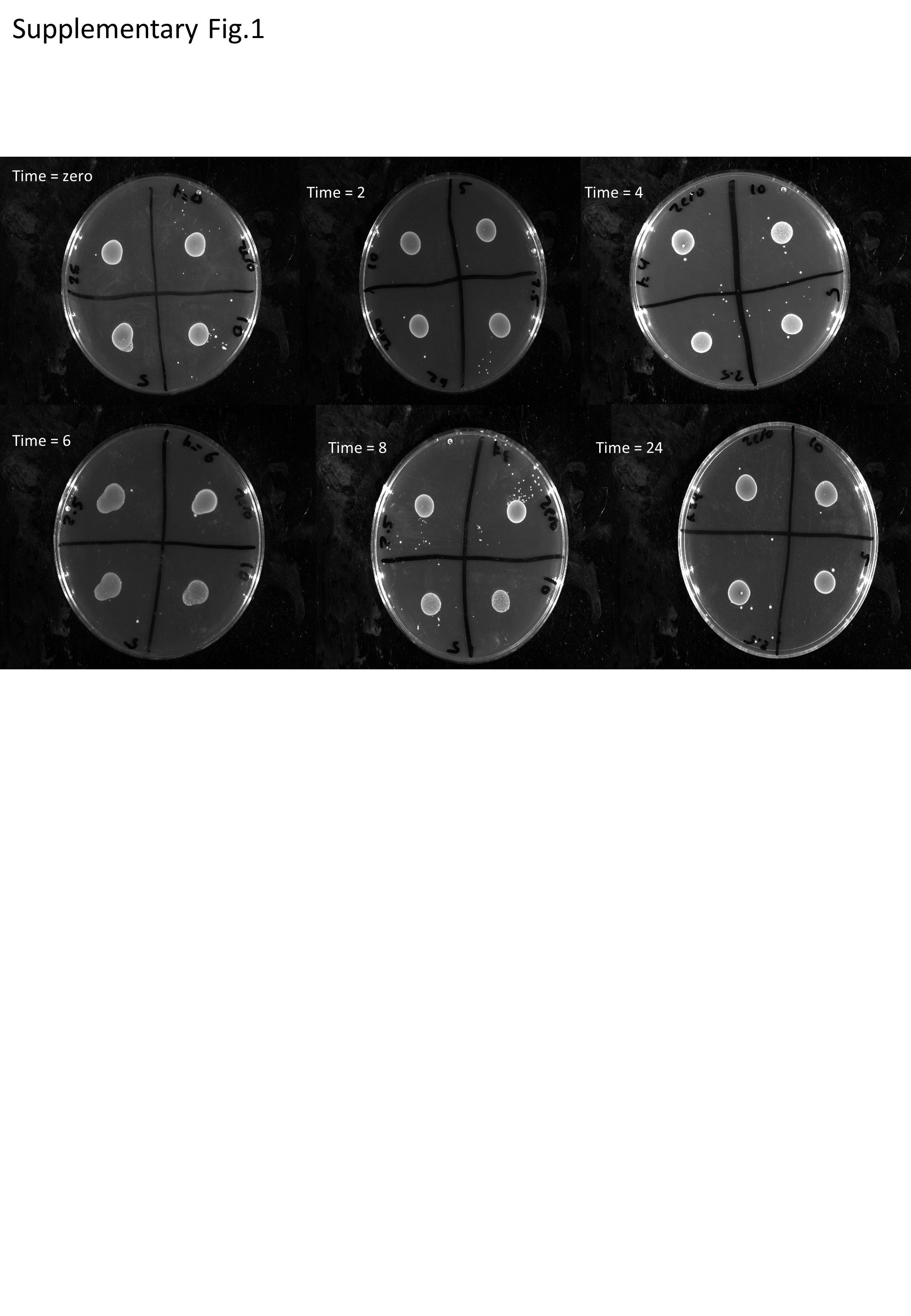

Supplement: Supplementary Figure 1 — Drop method to detect bacterial growth of S. mutans on BHI-agar plates after treatment with different concentrations of CBG (0–10 μg/ml) at various time points. At the end of incubation, 10 μl of each sample was applied in triplicates on BHI agar plates and incubated overnight at 37°C. [file Image_1.JPEG]

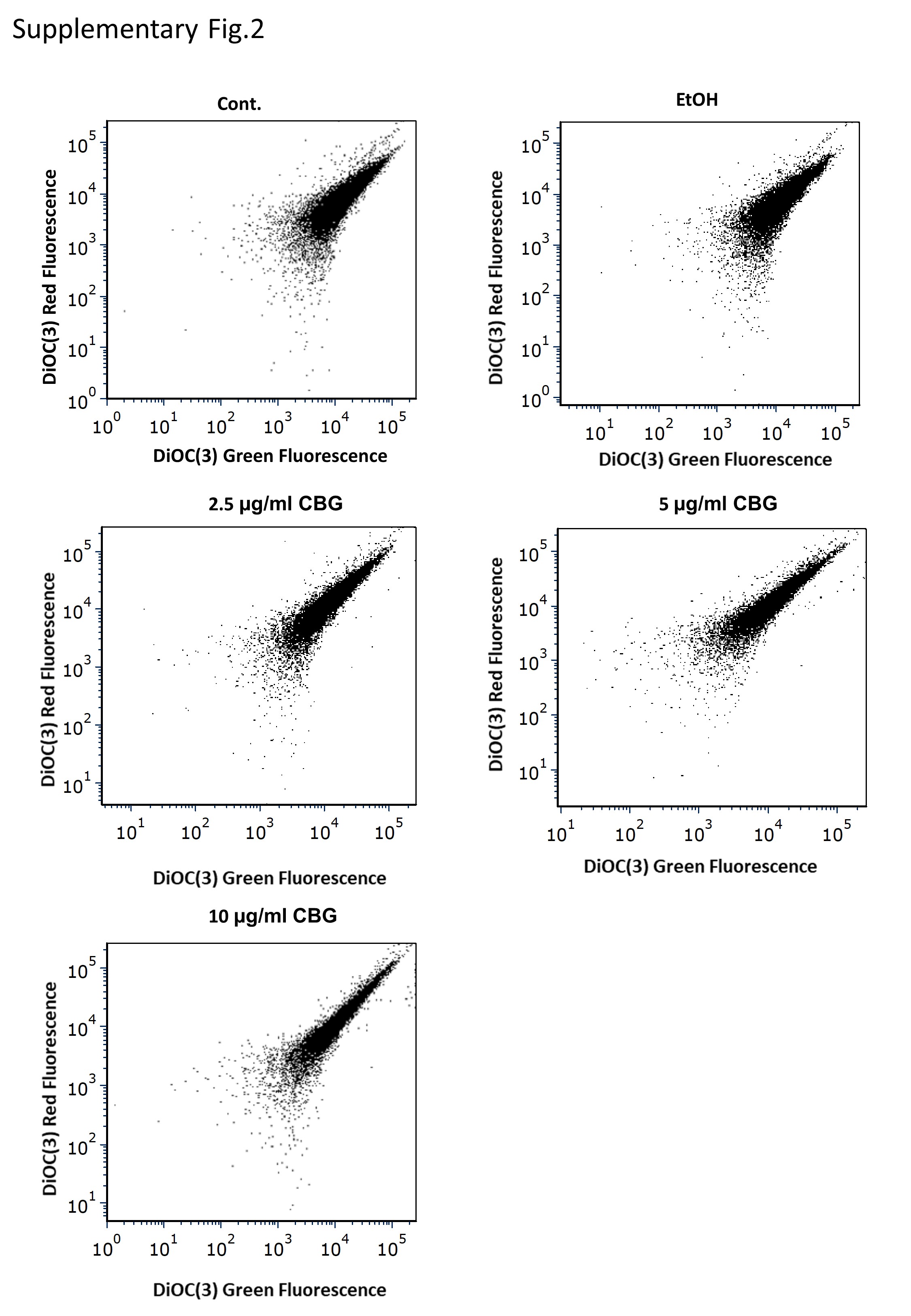

Supplement: Supplementary Figure 2 — Flow cytometry dot plots of DiOC2(3)-stained S. mutans that have been exposed to different CBG concentrations (0–10 μl/ml) for 30 min. The Y-axis shows the red fluorescence, while the X-axis shows the green fluorescence. [file Image_2.JPEG]
